# Change of Form

**Published:** 1856-02

**Authors:** 


*Change of Form.*

*Nelson's Northern Lancet,* which was formerly a monthly medical journal, has been changed to a *weekly* issue, but it remains under the same editorial management.

We have now three *weekly* medical journals in the United States, viz: The "*Boston Medical and Surgical Journal,*" the "*The Medical Councelor,*" Columbus, Ohio, and the "*Northern Lancet.*"

